# Substitution of Pork Fat by Emulsified Seed Oils in Fresh Deer Sausage (‘Chorizo’) and Its Impact on the Physical, Nutritional, and Sensory Properties

**DOI:** 10.3390/foods12040828

**Published:** 2023-02-15

**Authors:** Elena Martínez, José Emilio Pardo, Manuel Álvarez-Ortí, Adrián Rabadán, Arturo Pardo-Giménez, Andrés Alvarruiz

**Affiliations:** 1 Escuela Técnica Superior de Ingeniería Agronómica y de Montes y Biotecnología, Universidad de Castilla-La Mancha, Campus Universitario, s/n, 02071 Albacete, Spain; 2 Centro de Investigación, Experimentación y Servicios del Champiñón (CIES), C/Peñicas, s/n, Quintanar del Rey, 16220 Cuenca, Spain

**Keywords:** processed meat products, seed oils, functional food, pork fat replacement, consumer preference, sausage formulation

## Abstract

Meat products are consumed worldwide, but their high content of saturated fatty acids requires a reformulation of that type of food. In this regard, the objective of this study is to reformulate ‘chorizos’ by replacing the pork fat with emulsified seed oils from seeds (50%, 75%, and 100%). Commercial seeds (chia and poppy) and other seeds considered wastes from the agri-food industry (melon and pumpkin) were evaluated. Physical parameters, nutritional composition, fatty acid profile, and consumer evaluation were analyzed. The reformulated chorizos presented a softer texture but a better fatty acid profile due to their decrease in saturated fatty acids and their increase in linoleic and linolenic fatty acids. Regarding consumer evaluation, all the batches were positively evaluated in all the parameters studied.

## 1. Introduction

Consumers are nowadays more concerned about the impact of food on their health since many factors that, include development in health education and healthier lifestyles, have positively impacted consumer preferences, as they demand healthier and more natural food products [1]. Due to the recent developments in food science, the food industry can provide innovative methods that can modify the physical structure and the chemical composition of food products [2].

With these new advances, it is possible to introduce new components into a food product to make it healthier or substitute an element that is harmful to health to prevent the risk of certain diseases.

Meat is an important food for humans due to its high-quality protein content, but excessive consumption of meat products, more specifically processed meat products, is not recommended due to their high levels of sodium and animal fat [3]. The fat used in the formulation of processed meat products contains more saturated fatty acids than unsaturated fatty acids [4]. The food industry is facing a great challenge in searching for new formulations that replace saturated fatty acids [5]. Nevertheless, fats and oils are essential components in the diet that are used in food products due to their nutritional value, their texture, and sensory properties.

The presence of many bioactive compounds in meat makes meat products a great vehicle to introduce these compounds into the diet without changing consumers’ habits, and due to those benefits, there has been an important development of functional meat products [6]. Animal fat replacement by vegetable oils has gained importance in the last recent years because of the benefits conferred on the final products [7]. Products such as nuts and seeds contain large quantities of monounsaturated and polyunsaturated fatty acids [8], but the inclusion of vegetable oils in food products is not always technologically feasible due to their flavor, color, and fatty acid composition [9].

Therefore, many researchers have developed studies related to the emulsification of vegetable oils by using techniques such as microencapsulation with maltodextrin, alginate, and cyclodextrin or the creation of a gel net using gelling agents (i.e., konjac or alginate) [10,11]. Vegetable oil emulsions have been applied in the reformulation of processed meat products such as burgers or sausages, which has a positive impact on the lipidic profile and content but affects, in some cases, the sensorial and texture parameters, resulting in softer products that present lower intensities in all the flavor attributes when using inulin and citrus fiber as fat replacers [12] In this study, maltodextrin was used due to its easy availability, low cost, and simplicity of emulsion preparation.

The intake of adequate quantities of linolenic and linoleic fatty acids in the diet is fundamental for the prevention and treatment of some diseases, such as cancer, cardiovascular diseases, and metabolic or immune pathologies. Particularly, chia seeds are a suitable source of polyunsaturated fatty acids, mainly omega-3 (54–67%) and omega-6 (12–21%) [13]. Poppy seeds are rich in fat, the predominant fatty acid, linoleic acid (53–74%) [14]. Melon and pumpkin seed oil present high proportions of linoleic fatty acid (52–69%; 35.6–60.8%) [15,16]. In addition, they are considered food waste from the agri-food industry, which is an added value in the frame of the circular economy.

Deer meat presents numerous benefits compared to other meats. It is rich in mineral salts, essential amino acids, vitamins, and proteins, and it is low in fat [17]. It has traditionally been used for consumption around Europe, and nowadays, wild game meat responds to actual consumers’ trends in terms of the sustainability of meat production and consumption [18].

Therefore, based on the new trends of nutritional improvement of food products, the present study aims to substitute saturated fat from pork fat by using emulsified seed oils in the formulation of fresh deer meat chorizo. Pork fat was used instead of deer meat because the content of fat in deer meat is less than 2%; moreover, pork fat adds juiciness to the final product. Chia, poppy, melon, and pumpkin seed oils were used in this study. Physical, nutritional, and sensory properties were examined to evaluate the reformulated sausages compared to the control sample.

## 2. Materials and Methods

### 2.1. Ingredients and Chorizo Formulation

Deer meat, pork jowl, and pork tripe were obtained from a market specializing in game meat located in Los Yébenes (Toledo, Spain). Condiments and additives needed for chorizo formulation were obtained from Sosa S.L. (Cataluña, Spain). Deer meat was used as the main component of the sausages, pork jowl was added as fat to increase the juiciness of burgers, and pork tripe was used to stuff the sausages. 

Melon (Cucumis melo L.) and pumpkin (Cucurbita maxima) seeds were obtained from Vicente Peris S.A. (Valencia, Spain). The first steps to prepare the seeds consist of cleaning and washing them to remove any sugar or residue present on pumpkin and melon seeds. Then, as they presented a high moisture content, they were dried at room temperature until reaching a moisture content below 10%. Poppy (Papaver somniferum) and chia (Salvia hispanica) seeds were acquired in local supermarkets. The oils were obtained by using a hydraulic press (Mecamaq DEVF 80, Vila-Sana, Lleida, Spain). Seeds were grounded, and then they were subjected to a pressure of 200 bar for 10 min. After pressing, to remove any remaining solid, oil was centrifuged and then stored in opaque glass bottles at refrigerated temperature (4 °C ± 2 °C) to avoid deterioration.

For this experiment, a control sample and twelve formulations with different levels of fat substitution and oils were developed. The first step consisted of preparing the oil emulsions by using 22 g of maltodextrin per 100 mL of oil. Oil and maltodextrin were mixed using a spoon and kept at 4 °C ± 2 °C for 24 h.

In the reformulation of the chorizos, deer meat and condiment content remained constant. For meat, the percentage was 60% of the total of the formula. As condiments (5% of the total recipe), paprika (60%), salt (10%), pepper (10%), oregano (10%), and garlic powder (10%) were used. Table 1 shows the percentages of pork fat and oil emulsion used in each reformulation.

For chorizo preparation, all the ingredients were weighed and mixed manually for 5 min. The mixture was placed in a stuffer and introduced in natural casings [19]. Chorizos were aired for 24 h and then packed and stored at 4 °C until carrying out the evaluations. 

### 2.2. Physical Analysis

Color results were obtained by using a Minolta CR-200 colorimeter (Minolta Camera Co., Ltd., Osaka, Japan) with a D65 illuminant by measuring 4 different points on the surface of 4 different whole fresh chorizos of each batch. The CIElab chromatic coordinates: L* (lightness), a∗ (red-green component), b* (yellow-blue component) were calculated from the tristimulus values [20].

The texture profile analysis (TPA) test was used to evaluate the differences in texture. The analysis was carried out with a TA-TX Plus (Stable Micro Systems, Godalming, U.K.). For each type of cooked chorizo, 4 samples consisting of 2 cm thick slices were analyzed. The samples were placed horizontally and were subjected to 2 consecutive compression cycles simulating the chewing process.

Moisture content was quantified according to the ISO standards 1442: 1997 [21]. Cooking losses were determined by pre-weighting 4 fresh chorizo pieces taken from each batch before cooking. Samples were grilled in a pan until reaching a core temperature of 71 °C. After cooling for 5 min, the chorizos were weighted again, and weight loss was calculated as g/100 g.

### 2.3. Proximate Analysis

To obtain ash content results, samples were calcinated at 550 °C until reaching constant weight [22]. For the protein content, the Kjeldahl method was applied by multiplying the total nitrogen content by a conversion factor of 6.25 [23]. Crude fat was obtained by the use of the filter bag technique in an Ankom XT10 extractor (ANKOM Tecnology, Macedon, NY, USA) [24]. On the other hand, crude fiber content results were retrieved by using the Weende technique adapted to the filter bag technique as described by ANKOM [23], in which the organic residue is determined from digestion with solutions of sodium hydroxide and sulfuric acid in an Ankom 220 fiber analyzer (ANKOM Technology, Macedon, NY, USA).

The total carbohydrate content was calculated by detracting the sum of protein, fat, water, and ash from the total carbohydrate content [25]. Finally, total energy was calculated from the bases of the Atwater values, which are for fat (9 kcal/g), protein (4 kcal/g), and carbohydrate (4 kcal/g), based on 100 g of the sample [26].

### 2.4. Fatty Acid Profile

The fatty acid profile was obtained by gas chromatography in a Shimadzu GC-2010 Plus Gas Chromatograph (Shimadzu, Tokyo, Japan) using a CPSil 88 fused silica capillary column (50 m × 0.25 mm i.d.), 0.20 m film thickness (Varian, Middelburg, The Netherlands), and helium as the carrier gas (120 kPa). Each fatty acid methyl ester (FAME) was identified by direct comparison with a standard mixture (FAME 37, Supelco, Bellefonte, PA, USA) [27]. Two samples of each batch were analyzed after the extraction of the fat, and the results are expressed as the percentage of each FAME. On the other hand, Atherogenicity (AI) was calculated from the percentage of lauric (C12:0), myristic (C14:0), and pamitic (C16:0) acids, and the sum of the polyunsaturated (PUFA) and monounsaturated (MUFA) fatty acids. On the other hand, the Thrombogenicity index (TI) was calculated by using the percentage of myristic, palmitic, and stearic (C18:0) acids and the sums of MUFA, omega 6 (*n* = 6) and the ratio between omega-6 and omega-3 (*n* = 3).

(1) $$\mathrm{AI}=\frac{C12:0+\left(4*C14:0+C16:0\right)}{\sum^{\;}\mathrm{PUFA}+\sum^{\;}\mathrm{MUFA}}$$

(2) $$\mathrm{TI}=\frac{C14:0+C16:0+C18:0}{0.5*\sum^{\;}\mathrm{MUFA}+0.5\sum^{\;}\left(n=6\right)+3\sum^{\;}\left(n=3\right)+\frac{\sum^{\;}\left(n=3\right)}{\sum^{\;}\left(n=6\right)}}$$

### 2.5. Consumer Evaluation

To measure the acceptance of consumers for the different chorizos’ formulas, an affective test was carried out. A total of 101 consumers were selected among the staff and students at the University of Castilla-La Mancha, who evaluated a total of 13 chorizo batches. For the external aspect, chorizos were evaluated fresh, while for odor, taste, and texture, samples were cooked until reaching 70 °C on the inside part of the chorizo and then sliced into small portions. In addition, mineral water and unsalted crackers were provided to cleanse the palate between samples [28]. During the evaluation, consumers were asked to rate on a 9-point scale how much they liked each of the samples, being −4: dislike extremely, 0: neither like nor dislike, +4: like extremely. The time spent by each consumer was around 25 min.

### 2.6. Statistical Analysis

Results are expressed as mean values ± standard deviation (SD). A total of 4 chorizo samples from each batch were analyzed for the physical and nutritional evaluation, and 2 for the fatty acid parameters. For the sensory evaluation, the scores of 101 consumers were used. The analysis of variance (ANOVA) test was used to calculate the statistical differences, using Duncan’s multiple range test as a post hoc comparison of statistical (version 28.0).

## 3. Results

### 3.1. Physical Analysis

Cooking loss determines the capacity of the system to tie water and fat after protein denaturation and aggregation [29]. Table 2 shows the values obtained for cooking loss, moisture of cooked samples, and moisture retention.

In the present study, the lowest cooking loss was headed by the control sample, and this loss increases as the percentage of animal fat are reduced, so samples that contain 100% oil emulsion in the reformulation present the highest cooking loss.

Many studies have shown that lipid reformulation by using a gel matrix can reduce considerable cooking loss and fat binding capacity [30], but this is not the case in the present work. Note that we only used maltodextrin and oil to stabilize the emulsion, which may have caused bigger cooking loss ratios because the added maltodextrin resulted in an increase in the total expressible fluid, which caused a decrease in the stability of the emulsion. A study [31] carried out on reduced-fat beef sausages obtained similar results related to the cooking loss, which indicated that when using a commercial fiber, cooking loss increased in sausages, which indicated the weakness of this fiber in strengthening the ground meat mixture, which can also explain the higher cooking loss in the reformulated chorizos.

Nevertheless, moisture content decreases as the percentage of animal fat replacement increases. This is related to the emulsion employed, as it is just formed by oil and maltodextrin, a bulking agent, so the moisture content is not the same as that contained in the pork fat or in other emulsions where water is used.

One of the most important parameters that affect consumers’ choices when purchasing foods in color. Table 3 shows color parameters results (lightness (L *), redness (a *), and yellowness (b *)).

There are many factors, such as the type and quantity of pigment, the water content, and the extracts content, among others, that can affect lightness in food [32]. Moreover, as regards the color, the L * values were higher in the control sample compared to the reformulated samples, which means that lightness was affected by the animal fat content and the presence of emulsified seed oils [33]. Previous studies have shown that the color of processed meat products reformulated with vegetable oil was altered because of some modifications in the structure during the chopping process when the oil phase is distributed within the actomyosin matrix, which increases the surface of the fat particles [33]. Therefore, the replacement of animal fat results in darker chorizos.

Secondly, a * and b * parameters were also affected by the reformulation of fresh chorizos. The red color (a* ) of meat products is an important parameter that affects consumer preference and may be helpful in explaining its stability [34].

Control and melon 50% obtained the highest yellowness (b *). For the a * parameter, the control sample, melon 75% and melon 100% presented the lowest redness (a *) values, which means that they are less red and less stable compared with the rest of the samples and differ significantly from the rest of the reformulated chorizos, which are in line with the results obtained by [35]. Yellowness decreased with fat substitution compared to the control sample because of the presence of maltodextrin in the formulation and the pigments contained in the pumpkin, chia, and poppy oils [36].

Generally, the viscoelastic properties and the capacity to retain the water of the protein gels are linked to the hardness of meat products [37]. In this study, control samples presented the highest values for hardness. For increasing vegetable oil content, the chorizos presented softer textures (Table 4). The results concur with other similar studies in which the addition of oil emulsions reduces the hardness of meat products [37,38]. In addition, other authors reported an increase in all the parameters in reformulated samples, relating this phenomenon to the increase in protein–protein and protein–lipid interaction due to the lower fat globules of vegetable oils [39]. The percentage of substitution of animal fat, the type of vegetable oil used, as well as the method of oil emulsion can explain the differences between studies.

There were no significant differences in cohesiveness or elasticity. On the other hand, chewiness was lower as the animal fat content decreased, which means that it will be easier to chew samples that contain more percentage of vegetable oil.

### 3.2. Nutritional Analysis

Table 5 shows the results of the nutritional evaluation of the chorizo samples. There are no significant differences in nitrogen, ash, and protein content. Nevertheless, there are differences in crude fat, total carbohydrate content, and energy value.

As the seed oil content increases in the reformulation, the crude fat content decreases considerably compared to the control sample, which yielded the highest crude fat [38].

Total carbohydrates differ due to the use of tapioca maltodextrin for the emulsion of the oils. Therefore, as the content of oil increases, the total carbohydrate content also increases.

As reported by [40], the energy value was significantly reduced in the reformulated samples because of the reduction in the fat content.

### 3.3. Fatty Acid Profile

The aim of this study was to increase the fatty acid quality of chorizos. Regarding the lipid content, the reformulation with maltodextrin emulsion had a positive effect on the total fatty acid profile. In Table 6, only fatty acids that accounted for more than 5% are shown, although all the fatty acids detected have been considered for the calculation of MUFA, PUFA, AI, and TI.

The type of vegetable oil has an impact on the fatty acid composition of the reformulated products. These oils are rich sources of MUFAs and PUFAs and are cholesterol-free. In addition, vegetable oils have been used because of their content of other bioactive compounds, some of them presenting potential antioxidant activity [41].

Oleic, palmitic, and stearic acids make up 93% of the total fatty acids identified in control chorizos, which was also reported by [42].

Depending on the type of seed and substitution percentage, different types of fatty acids became more abundant. Poppy oil is rich in linoleic acid but also contains pro-health components such as phytosterols, tocopherols, and phenolics [14]. Chia stands out for being a source of omega-3 fatty acids and antioxidants such as chlorogenic and caffeic acids [42]. Melon and pumpkin seeds are rich in linolic acid, but pumpkin seed oil is also rich in vitamins, minerals, and carotenoids, and melon seed oil is rich in tocopherols, phospholipids, and sterols [43].

The addition of poppy, pumpkin, and melon oil, reduces the content of palmitic, stearic, and oleic acid while the linoleic acid increases. In the other samples, the increase in linoleic acid content is less dramatic but nevertheless remarkable. On the other hand, the samples reformulated with chia seed oil present the highest values for linolenic acid, differing significantly from the rest of the samples (*p* < 0.05).

Monounsaturated fatty acid content (MUFA) was lower for the reformulated chorizos compared with the control sample (Table 7). The polyunsaturated fatty acid fraction (PUFA) increased instead notably in the reformulated chorizos, being higher as the replacement of animal fat increased. This increase is directly related to linoleic and linolenic fatty acid contents in vegetable oils.

In our study, the atherogenic index (AI) and thrombogenic index (TI) were calculated. A reduction in both indexes was noted when the pork fat was substituted by the seed oils. AI decreased by about 50% when 100% of pork fat was substituted 100%, while the TI was in 100% oil-substituted chorizos, depending on the used seed. This is a very interesting result since these indexes are strong markers for the prediction of the risk of atherosclerosis, coronary heart disease, and/or thrombus formation [44].

Finally, lipid reformulation confers benefits for the marketing of meat products because health claims, such as ‘high in omega-3 fatty acids’ or ‘reduced lipid content’, could be included on their label [45].

### 3.4. Consumer Evaluation

**Figure 1:**
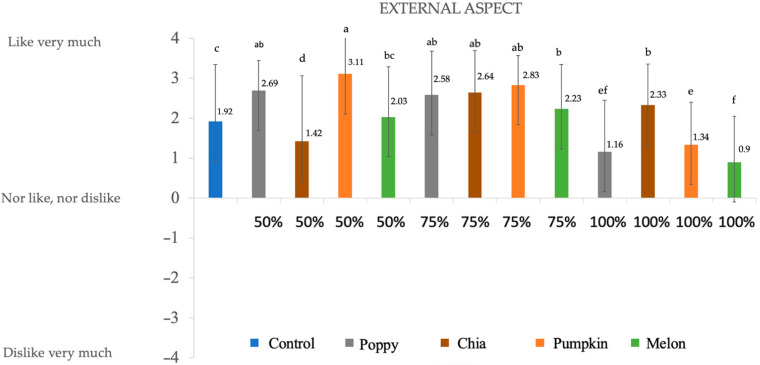
Results obtained in the sensory evaluation for external aspect. Numbers are mean ± standard error. Different letters in the same column for each attribute indicate statically differences (*p* < 0.05).

**Figure 2:**
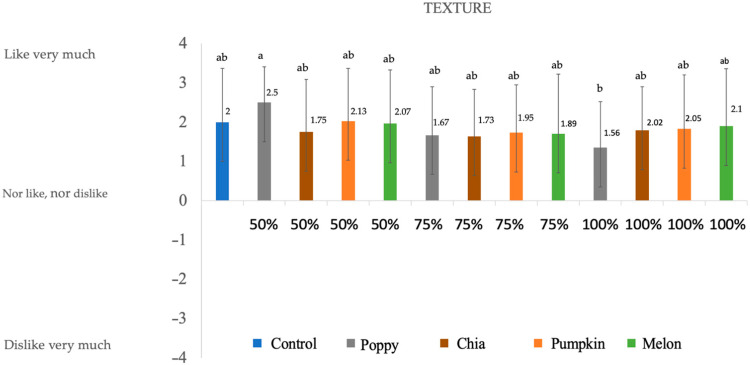
Results obtained in the sensory evaluation for texture. Numbers are mean ± standard error. Different letters in the same column for each attribute indicate statically differences (*p* < 0.05).

**Figure 3:**
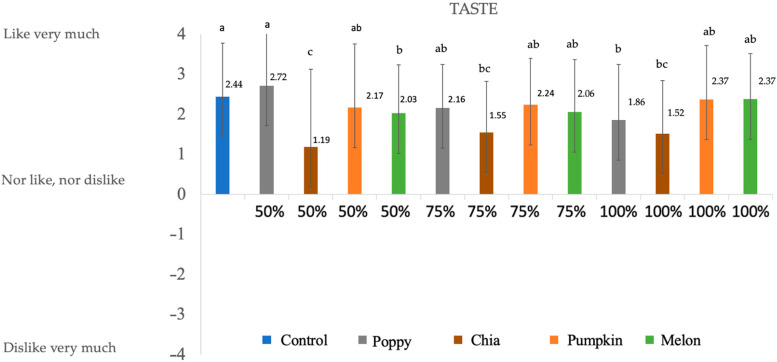
Results obtained in the sensory evaluation for taste. Numbers are mean ± standard error. Different letters in the same column for each attribute indicate statically differences (*p* < 0.05).

**Figure 4:**
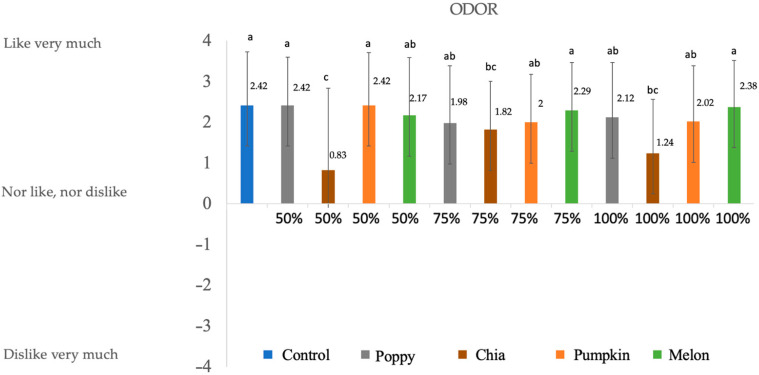
Results obtained in the sensory evaluation for odor. Numbers are mean ± standard error. Different letters in the same column for each attribute indicate statically differences (*p* < 0.05).

The sensory properties of deer chorizos (Figure 1, Figure 2, Figure 3 and Figure 4) were influenced by the reformulation with seed oils. Since fat contributes to the color, texture, and taste of meat products, reducing or substituting animal fat in the chorizo formulation may alter the sensory perception. Note that all the batches present positive values for all the evaluated parameters.

Regarding the texture of the chorizos, there are no big differences between the samples. The lowest value for texture was poppy 100%. This means that although they present a softer texture (TPA) compared with the control sample, they were nevertheless well accepted, as values are above 0 in all cases.

No significant differences were found for odor (*p* < 0.5) among the control sample, poppy 50%, pumpkin 50%, melon 75%, and melon 1000%. For the rest of the samples, scores are above 0, and in most of the cases (melon 50%, pumpkin 75%, poppy 100%, and pumpkin 100%) were valued as ‘I like a lot’. A study carried out on fermented sausages did not show big differences among samples for odor when adding olive oil in the reformulation [46]. On the other hand, the worst valued was chia 50%, followed by chia 75% and chia 100%, which may be due to the large quantities of Omega-3 present in the oil, which produces a fishy odor when heated.

All the batches obtained positive scores for taste. Control sample and poppy 50% obtained the highest scores, but they do not differ significantly from the reformulated samples with melon and pumpkin oil (50%, 75%, and 100%). Other studies [47] found that the replacement of animal fat with vegetable oils did not affect the taste of deer burgers and came to the conclusion that the greater the PUFA content, the lower was valued the taste parameter. Our results agree with this study, as poppy 100% and chia (50%, 75%, and 100%) contain higher percentages of PUFAs and are also less valued. Our findings also agree with the results reported by [7,47], who stated that burgers reformulated with chia oil were correlated with negative descriptors for taste, which was perceived as unpleasant. Chia samples also obtained the lowest scores for odor. It appears that the use of chia oil in the reformulation of meat products reduces the sensory quality [48]. The inclusion of chia oil in the form of hydrogelled emulsion could increase the acceptability of chia samples [49].

## 4. Conclusions

This study demonstrates the possibility of replacing animal fat with emulsified seed oils to obtain fresh chorizos with a better fatty acid profile where linolenic and linoleic fatty acids replace saturated fatty acids. Furthermore, the results show an important decrease in the fat content as the percentage of animal fat replaced increases, which directly affects the energy value.

The addition of emulsified oils results in a decrease in the hardness of the reformulated samples compared to the traditional ones. Furthermore, the presence of emulsified oils also affects the color, being the reformulated samples darker and more red.

Regarding consumer evaluation, all the batches have been valued positively, but in general, in all the parameters analyzed, chia samples obtained the lowest values due to their odor and flavor.

## Figures and Tables

**Table 1:** Percentage of animal fat substitution used in each sample.

Sample	Pork Fat	Oil Emulsion
Control	35%	0%
Poppy 50%	17.5%	17.5%
Chia 50%	17.5%	17.5%
Pumpkin 50%	17.5%	17.5%
Melon 50%	17.5%	17.5%
Poppy 75%	8.75%	26.25%
Chia 75%	8.75%	26.25%
Pumpkin 75%	8.75%	26.25%
Melon 75%	8.75%	26.25%
Poppy 100%	0%	35%
Chia 100%	0%	35%
Pumpkin 100%	0%	35%
Melon 100%	0%	35%

**Table 2:** Results obtained for cooking loss, moisture of cooked samples, and moisture retention in fresh deer chorizos.

Sample	Cooking Loss (%)	Moisture Cooked (%)	Moisture Retention (%)
Control	2.88 ± 0.41 ^g^	56.83 ± 0.04 ^a^	55.19± 0.18 ^a^
Poppy 50%	10.66 ± 0.20 ^f^	53.47 ± 0.74 ^a,b^	47.76 ± 0.51 ^b^
Chia 50%	9.48 ± 0.58 ^f^	54.95 ± 1.35 ^a,b^	48.45 ± 0.39 ^b^
Pumpkin 50%	11.05 ± 0.08 ^f^	54.72 ± 0.38 ^a,b^	48.68 ± 0.29 ^b^
Melon 50%	11.08 ± 0.78 ^f^	52.95 ± 1.34 ^b^	47.08 ± 1.61 ^b^
Poppy 75%	17.16 ± 0.93 ^c,d,e^	50.39 ± 0.27 ^c,d^	41.75 ± 0.72 ^a^
Chia 75%	17.42 ± 0.13 ^c^	50.90 ± 1.55 ^c^	42.02 ± 1.21 ^b,c^
Pumpkin 75%	15.14 ± 0.13 ^e^	50.14 ± 0.49 ^d^	42.06 ± 0.13 ^b,c^
Melon 75%	16.10 ± 0.17 ^d^	51.00 ± 0.70 ^c^	43.28 ± 0.66 ^c^
Poppy 100%	24.29 ± 0.86 ^a^	45.37 ± 1.07 ^e^	34.30 ± 0.42 ^f^
Chia 100%	19.19 ± 0.05 ^b,c^	48.00 ± 1.41 ^d,e^	38.78 ± 1.11 ^d^
Pumpkin 100%	18.08 ± 0.27 ^b,c,d^	48.01 ± 0.01 ^d,e^	38.38 ± 0.05 ^e^
Melon 100%	20.03 ± 0.08 ^b^	47.24 ± 1.75 ^d,e^	38.70± 1.57 ^d^

Numbers are mean ± standard error. Different letters in the same column for each attribute indicate statically differences (*p* < 0.05).

**Table 3:** Results obtained for the color parameters of the chorizos prepared with different emulsified seed oils.

Sample	L *	a *	b *
Control	42.16 ± 1.12 ^a^	9.83 ± 0.43 ^c^	16.94 ± 0.73 ^a^
Poppy 50%	37.95 ± 1.98 ^b,c^	11.19 ± 0.94 ^b^	11.85 ± 1.03 ^c^
Chia 50%	34.80 ± 1.50 ^c^	11.91 ± 0.25 ^b^	12.56 ± 1.04 ^c^
Pumpkin 50%	32.61 ± 1.59 ^d^	16.42 ± 1.34 ^a^	11.26 ± 1.15 ^c^
Melon 50%	38.62 ± 1.25 ^b^	10.53 ± 0.49 b ^b,c^	16.82 ± 0.98 ^a^
Poppy 75%	37.04 ± 1.18 ^b,c^	11.63 ± 1.38 ^b^	13.64 ± 1.19 ^b^
Chia 75%	36.65 ± 1.19 ^b,c^	9.61 ± 1.41 ^c^	11.33 ± 1.48 ^c^
Pumpkin 75%	35.09 ± 0.78 ^c^	14.03 ± 1.22 ^a,b^	14.08 ± 1.31 ^b^
Melon 75%	37.13 ± 1.35 ^b,c^	9.43 ± 0.66 ^c^	12.69 ± 1.17 ^b,c^
Poppy 100%	37.19 ± 0.96 ^b,c^	10.89 ± 0.82 ^b^	11.87 ± 1.11 ^c^
Chia 100%	36.33 ± 1.74 ^b,c^	10.15 ± 1.46 ^b,c^	13.24 ± 3.04 ^b^
Pumpkin 100%	36.89 ± 1.67 ^b,c^	11.96 ± 0.26 ^b^	14.74 ± 2.16 ^b^
Melon 100%	35.23 ± 1.73 ^c^	9.51 ± 0.28 ^c^	10.69 ± 0.28 ^d^

Numbers are mean ± standard error. Different letters in the same column for each attribute indicate statically differences (*p* < 0.05).

**Table 4:** Results obtained for the texture parameters analyzed in the different reformulated samples.

Sample	Hardness (N)	Cohesiveness (Ratio)	Elasticity	Chewiness (N)
Control	419.95 ± 1.87 ^a^	0.41 ± 0.08	0.44 ± 0.04	668.36 ± 1.80 ^a^
Poppy 50%	261.38 ± 2.82 ^d^	0.39 ± 0.03	0.39 ± 0.05	405.88 ± 1.22 ^b,c^
Chia 50%	294.24 ± 1.86 ^c^	0.39 ± 0.03	0.43 ± 0.04	495.53 ± 1.71 ^b^
Pumpkin 50%	347.46 ± 2.89 ^b^	0.39 ± 0.01	0.45 ± 0.12	498.63 ± 1.12 ^b^
Melon 50%	336.26 ± 1.76 ^b^	0.40 ± 0.06	0.42± 0.14	356.12 ± 2.50 ^c,d^
Poppy 75%	244.53 ± 1.40 ^e^	0.39 ± 0.15	0.42 ± 0.11	339.35 ± 3.53 ^d^
Chia 75%	275.59 ± 1.49 ^c,d^	0.40 ± 0.05	0.41 ± 0.09	305.74 ± 4.48 ^d^
Pumpkin 75%	278.90 ± 6.70 ^c,d^	0.43 ± 0.02	0.42 ± 0.16	381.57 ± 2.03 ^c^
Melon 75%	264.33 ± 5.59 ^d^	0.42 ± 0.05	0.41 ± 0.06	212.62 ± 3.16 ^f^
Poppy 100%	125.96 ± 5.42 ^f^	0.41 ± 0.12	0.39 ± 0.16	200.02 ± 2.26 ^g^
Chia 100%	120.13 ± 1.32 ^f^	0.41 ± 0.04	0.40 ± 0.07	236.40 ± 3.81 ^e^
Pumpkin 100%	197.23 ± 2.66 ^e^	0.42 ± 0.03	0.41 ± 0.11	218.32 ± 4.28 ^f^
Melon 100%	117.42 ± 3.49 ^g^	0.40 ± 0.05	0.39 ± 0.13	260.67 ± 4.14 ^e^

Numbers are mean ± standard error. Different letters in the same column for each attribute indicate statically differences (*p* < 0.05).

**Table 5:** Results obtained for the nutritional parameters analyzed in the different reformulated samples.

Sample	Nitrogen (%)	Protein (%)	Ashes (g/100 g)	Crude Fiber (g/100 g)	Crude Fat (g/100 g)	Total Carbohydrates (g/100 g)	Energy Value (kcal/100 g)
Control	6.09 ± 0.055	38.03 ± 0.77	4.10 ± 1.04	2.48 ± 0.12 ^a,b^	47.07 ± 0.05 ^a^	10.77 ± 0.81 ^d^	618 ± 0.76 ^a^
Poppy 50%	6.81 ± 0.012	38.75 ± 0.20	4.63 ± 1.36	1.57 ± 0.21 ^c^	32.12 ± 0.09 ^c^	11.48 ± 042 ^d^	530 ± 1.22 ^c^
Chia 50%	6.46 ± 0.10	40.40 ± 0.65	4.79 ± 1.34	1.56 ± 0.18 ^c^	37.08 ± 0.07 ^b,c^	14.72 ± 0.68 ^c,d^	554 ± 1.14 ^b,c^
Pumpkin 50%	7.44 ± 0.32	38.53 ± 2.01	5.32 ± 1.14	1.94 ± 0.46 ^b^	36.04 ± 0.05 ^b,c^	10.10 ± 2.08 ^d^	550 ± 1.14 ^b,c^
Melon 50%	6.07 ± 0.22	37.93 ± 1.41	4.75 ± 1.42	1.63 ± 0.28 ^b^	40.36 ± 0.03 ^b^	13.95 ± 1.38 ^c,d^	570 ± 1.85 ^b^
Poppy 75%	6.39 ± 0.26	38.96 ± 1.63	4.28 ± 1.24	2.64 ± 0.21 ^c^	32.14 ± 0.01 ^c^	23.60 ± 1.71 ^b^	543 ± 1.01 ^b,c^
Chia 75%	6.53 ± 0.40	40.84 ± 2.50	4.70 ± 1.25	2.82 ± 0.06 ^b,c^	31.30 ± 0.01 ^c^	23.15 ± 2.67 ^b^	537 ± 1.08 ^c^
Pumpkin 75%	6.79 ± 0.25	40.46 ± 1.62	4.21 ± 2.14	3.02 ± 0.11 ^a,b^	33.65 ± 0.06 ^c^	19.51 ± 1.65 ^b,c^	551 ± 0.71 ^b,c^
Melon 75%	7.62 ± 0.17	40.65 ± 2.25	5.37 ± 1.29	2.51 ± 0.26 ^a,b^	23.67 ± 0.08 ^d^	23.29 ± 2.09 ^b^	496 ± 0.91 ^d^
Poppy 100%	7.07 ± 0.17	40.23 ± 1.07	5.33 ± 1.07	3.07 ± 0.08 ^a,b^	12.91 ± 0.03 ^f^	37.51 ± 0.07 ^a^	443 ± 1.52 ^f^
Chia 100%	7.76 ± 0.22	40.51 ± 1.38	4.65 ± 1.38	3.41 ± 0.21 ^b^	19.61 ± 0.05 ^e^	26.21 ± 1.07 ^b^	475 ± 1.52 ^e^
Pumpkin 100%	7.19 ± 0.18	40.18 ± 1.12	4.46 ± 1.44	3.58 ± 0.17 ^a^	19.08 ± 0.01 ^e^	23.27 ± 1.55 ^b^	469 ± 1.80 ^e,f^
Melon 100%	6.57 ± 0.25	40.66 ± 1.58	4.87 ± 1.13	3.15 ± 0.07 ^b^	15.81 ± 0.10 ^e,f^	38.55 ± 1.76 ^a^	459 ± 0.81 ^e,f^

Numbers are mean ± standard error. Different letters in the same column for each attribute indicate statically differences (*p* < 0.05).

**Table 6:** Fatty acid composition of the different reformulated samples.

Sample	Palmitic Acid (%)	Stearic Acid (%)	Oleic Acid (%)	Linoleic Acid (%)	Linolenic Acid (%)
Control	24.53 ± 0.16 ^a,b^	13.65 ± 0.26 ^a^	44.05 ± 0.81 ^a^	10.53 ± 0.33 ^h^	0.70 ± 0.03 ^f^
Poppy 50%	20.23 ± 0.05 ^c^	11.05 ± 0.13 ^b^	33.61 ± 0.33 ^c^	29.14 ± 0.18 ^d^	0.69 ± 0.05 ^f^
Chia 50%	18.26 ± 0.31 ^d,e^	10.35 ± 0.09 ^b,c^	31.48 ± 0.34 ^d^	14.42 ± 0.60 ^g^	21.24 ± 0.14 ^c^
Pumpkin 50%	22.30 ± 0.18 ^b,c^	12.73 ± 0.28 ^a,b^	35.76 ± 0.38 ^b,c^	23.26 ± 0.15 ^e^	0.59 ± 0.04 ^g^
Melon 50%	18.56 ± 0.22 ^d^	9.53 ± 0.22 ^c^	37.67 ± 0.37 ^b^	30.02 ± 0.15 ^d^	0.53 ± 0.22 ^g^
Poppy 75%	16.64 ± 0.13 ^e^	8.73 ± 0.11 ^d^	28.50 ± 0.24 ^d,e^	41.49 ± 0.35 ^b,c^	0.83 ± 0.06 ^e^
Chia 75%	18.31 ± 0.04 ^d,e^	10.29 ± 0.04 ^b,c^	33.75 ± 0.35 ^c^	14.95 ± 0.10 ^g^	26.67 ± 0.25 ^b^
Pumpkin 75%	20.34 ± 0.08 ^c^	11.07 ± 0.11 ^b^	26.39 ± 0.29 ^e^	33.14 ± 0.19 ^d^	0.54 ± 0.06 ^g^
Melon 75%	17.86 ± 0.06 ^e^	12.43 ± 0.18 ^a,b^	34.03 ± 0.16 ^b,c^	29.44 ± 0.18 ^d^	0.53 ± 0.09 ^g^
Poppy 100%	13.32 ± 0.18 ^f^	9.16 ± 0.04 ^c^	20.17 ± 0.11 ^f^	54.58 ± 0.67 ^a^	1.08 ± 0.09 ^d^
Chia 100%	18.37 ± 0.13 ^d^	5.91 ± 0.11 ^e^	11.14 ± 0.06 ^g^	17.98 ± 0.22 ^f^	52.40 ± 0.42 ^a^
Pumpkin 100%	20.08 ± 0.08 ^c^	10.43 ± 0.13 ^b,c^	28.75 ± 0.95 ^d,e^	44.01 ± 0.09 ^b^	0.86 ± 0.06 ^e^
Melon 100%	14.79 ± 0.27 ^f^	12.14 ± 0.06 ^a,b^	26.39 ± 0.31 ^e^	39.26 ± 0.06 ^c^	0.38 ± 0.01 ^h^

Numbers are mean ± standard error. Different letters in the same column for each attribute indicate statically differences (*p* < 0.05).

**Table 7:** Results obtained for the health indices (MUFA, PUFA, AI, and TI).

Sample	PUFA (%)	MUFA (%)	AI (%)	TI (%)
Control	11.26 ± 0.33 ^j^	48.28 ± 0.81 ^a^	0.54 ± 0.15 ^a^	1.26 ± 0.81 ^a^
Poppy 50%	29.83 ± 0.15 ^h^	36.78 ± 0.35 ^d^	0.39 ± 0.02 ^c^	0.93 ± 0.35 ^b^
Chia 50%	35.66 ± 0.68 ^f^	33.89 ± 0.33 ^e^	0.32 ± 0.01 ^d^	0.33 ± 0.22 ^g^
Pumpkin 50%	23.86 ± 0.17 ^i^	38.81 ± 0.43 ^c^	0.46 ± 0.03 ^b^	1.12 ± 0.11 ^a,b^
Melon 50%	30.56 ± 0.16 ^h^	40.26 ± 0.42 ^b^	0.31 ± 0.01 ^d,e^	0.79 ± 0.01 ^d^
Poppy 75%	42.33 ± 0.31 ^d^	30.89 ± 0.06 ^f^	0.30 ± 0.01 ^e^	0.69 ± 0.04 ^e^
Chia 75%	41.63 ± 0.35 ^d^	28.36 ± 0.94 ^g^	0.40 ± 0.21 ^c^	0.29 ± 0.34 ^g^
Pumpkin 75%	29.98 ± 0.22 ^h^	35.90 ± 0.44 ^d^	0.38 ± 0.12 ^c^	0.99 ± 0.11 ^b^
Melon 75%	33.68 ± 0.18 ^g^	36.12 ± 0.14 ^d^	0.31 ± 0.09 ^de,^	0.72 ± 0.10 ^e^
Poppy 100%	55.67 ± 0.75 ^b^	23.74 ± 0.13 ^h^	0.24 ± 0.05 ^g^	0.56 ± 0.04 ^f^
Chia 100%	70.39 ± 0.63 ^a^	12.86 ± 0.10 ^i^	0.16 ± 0.05 ^h^	0.09 ± 0.06 ^h^
Pumpkin 100%	39.65 ± 0.07 ^e^	29.74 ± 0.92 ^f^	0.31 ± 0.09 ^d,e^	0.88 ± 0.09 ^c^
Melon 100%	44.86 ± 0.18 ^c^	28.09 ± 0.25 ^g^	0.27 ± 0.08 ^f^	0.69 ± 0.11 ^e^

Numbers are mean ± standard error. Different letters in the same column for each attribute indicate statically differences (*p* < 0.05).

## Data Availability

The data presented in this study are available on request from the corresponding author. The data are not publicly available due to privacy reasons.
